# Obesity impacts the regulation of miR-10b and its targets in primary breast tumors

**DOI:** 10.1186/s12885-019-5300-6

**Published:** 2019-01-18

**Authors:** Ari Meerson, Yaniv Eliraz, Hila Yehuda, Bridget Knight, Malcolm Crundwell, Douglas Ferguson, Benjamin P. Lee, Lorna W. Harries

**Affiliations:** 1 MIGAL - Galilee Research Institute, PO Box 831, 11016 Kiryat Shmona, Israel; 2 Tel Hai Academic College, Tel Hai, Israel; 3 Royal Devon and Exeter NHS Foundation Trust, Royal Devon and Exeter Hospital, Barrack Road, Exeter, UK; 4 University of Exeter Medical School, Barrack Road, Exeter, UK

**Keywords:** miR-10b, Breast cancer, Obesity, microRNA, Tumor suppressors, Oncogenes

## Abstract

**Background:**

Obesity increases breast cancer (BC) risk in post-menopausal women by mostly unknown molecular mechanisms which may partly be regulated by microRNAs (miRNAs).

**Methods:**

We isolated RNA from paired benign and malignant biopsies from 83 BC patients and determined miRNA profiles in samples from 12 women at the extremes of the BMI distribution by RNA-seq. Candidates were validated in all samples. Associations between miR-10b expression and validated target transcript levels, and effects of targeted manipulation of miR-10b levels in a primary BC cell line on proliferation and invasion potential, were explored.

**Results:**

Of the 148 miRNAs robustly expressed in breast tissues, the levels of miR-21, miR-10b, miR-451a, miR-30c, and miR-378d were significantly associated with presence of cancer. Of these, miR-10b showed a stronger down-regulation in the tumors of the obese subjects, as opposed to the lean. In ductal but not lobular tumors, significant inverse correlations were observed between the tumor levels of miR-10b and miR-30c and the mRNA levels of cancer-relevant target genes *SRSF1, PIEZO1, MAPRE1, CDKN2A, TP-53* and *TRA2B,* as well as tumor grade*.* Suppression of miR-10b levels in BT-549 primary BC–derived cells increased cell proliferation and invasive capacity, while exogenous miR-10b mimic decreased invasion. Manipulation of miR-10b levels also inversely affected the mRNA levels of miR-10b targets *BCL2L11, PIEZO1* and *NCOR2*.

**Conclusions:**

Our findings suggest that miR-10b may be a mediator between obesity and cancer in post-menopausal women, regulating several known cancer-relevant genes. MiR-10b expression may have diagnostic and therapeutic implications for the incidence and prognosis of BC in obese women.

**Electronic supplementary material:**

The online version of this article (10.1186/s12885-019-5300-6) contains supplementary material, which is available to authorized users.

## Background

Obesity and cancer are both major health problems in developed countries. While inherited and somatic mutations underlie tumorigenesis, the physiological micro- and macro-environment also affect it. Thus, obesity elevates the risk of multiple cancers, among them breast cancer (BC) [1–3], and several mechanisms underpinning this association have been proposed. Amongst these, chronic inflammation, higher secretion of leptin (accompanied by systemic leptin resistance [4]) and lower adiponectin levels in obese subjects may deregulate cell division and angiogenesis and promote the development of cancer [5, 6].

Although many genes are emerging as important links between obesity and cancer, more research is still needed to better understand the complexity of gene regulation that underpins this phenomenon. The expression of many genes is regulated by microRNAs (miRNAs), endogenous small non-coding RNAs (ncRNAs). MiRNAs bind to cis-elements in the 3′ untranslated region of specific target mRNAs and regulate their translation or stability; their levels are often dysregulated in disease states, especially in cancers and metabolic conditions [7, 8]. MiRNAs play an essential role in the physiology of metabolic processes, such as adipocyte differentiation, metabolic integration, insulin resistance and appetite regulation [9–11], and are often dysregulated in the tissues of obese animals and humans [10–12]. Several miRNAs that are associated with obesity also have known roles in carcinogenesis [13], and their deregulated expression may act as a functional link between obesity and cancer. Our previous study [14] has shown that the transcription factor ETS1, involved in multiple aspects of tumorigenesis and in many types of cancer [15], is a target of miR-221, and can function as an oncomiR or a tumor suppressor [16]. The oncogenic effects of miR-221 are also mediated by other downstream cell cycle regulators such as *CDKN1B*/p27 [17], *CDKN1C*/p57 [18], *PTEN*, *TIMP3* [19], and *PTPμ* [20]. Accordingly, miR-221 has been explored as a potential therapeutic target in different cancers [21, 22]. Similarly, we have previously shown that miR-4443 acts in a tumor-suppressive manner by down-regulating *TRAF4* and *NCOA1* downstream of MEK-C/EBP-mediated leptin and insulin signaling, and that insulin and/or leptin resistance (e.g. in obesity) may suppress this pathway and increase the risk of metastatic CRC [23]. These and other reports provide molecular evidence for the role of miRNAs in the chronic diseases of obesity and cancer; however, much more direct evidence establishing the precise role of specific miRNAs in obesity and cancer is still needed.

In this study, we characterized obesity-associated effects on miRNA expression changes in BC, using paired tumor and normal biopsies from 83 patients with a range of BMI, and identified a cancer-relevant miRNA (miR-10b) with levels significantly affected by obesity. We have also characterized the expression of several known cancer-relevant targets of this miRNA in the same samples and assessed the responses of this miRNA to upstream metabolic and inflammatory challenges in a cultured primary BC cell line (BT-549) in vitro. Finally, we determined the effects of manipulating miR-10b levels on cell kinetics and invasion potential in vitro. Identification of mediators between obesity and malignancy may help improve the diagnostics and personalize the treatment of metabolism-dependent cancers.

## Methods

### Cohort description

**Fig. 1:**
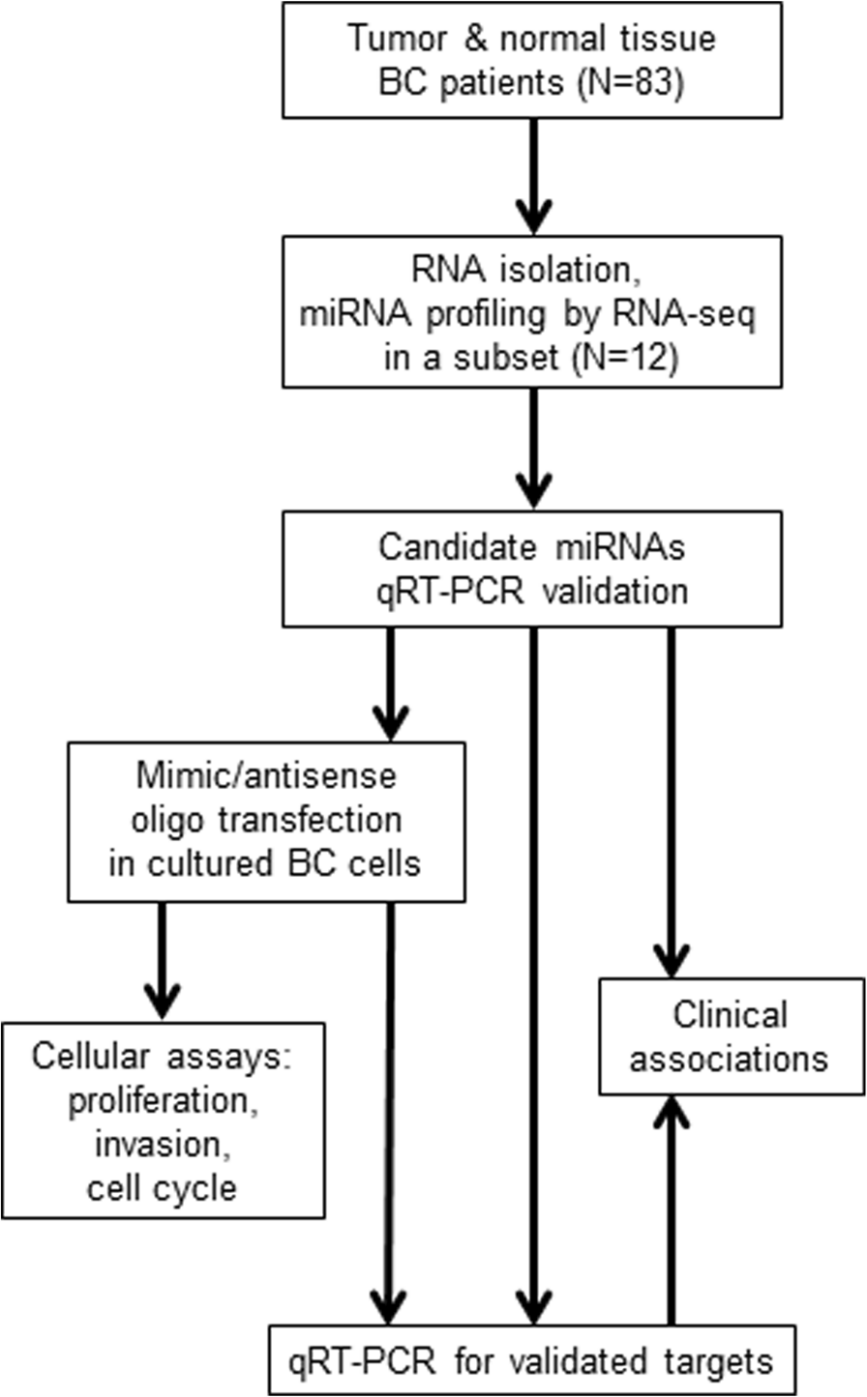
general scheme of the study

Eighty-three paired breast tissue samples (tumor + normal breast) were obtained from consenting patients during their standard cancer surgical treatment, with ethical permission from the NIHR Clinical Research Facility at the University of Exeter Medical School. The cohort consisted of female patients diagnosed with different subtypes and grades of BC, age range 39–84 (mean 67.75), BMI range 19–42 (mean 28). Samples were obtained following routine mastectomy and subjected to routine histological analysis. Both the tumor and surrounding tissues were removed in the mastectomy. Malignant tissue was obtained from cores of the tumor, and benign tissue was obtained from the normal tissue, remote from the tumor, in the mastectomy specimen. Tissue was categorized as normal or tumor based on histological examination. Further details of the patient cohort and the sample characteristics are presented in Table 1. Of these, 12 (6 with BMI > 31 and 6 with BMI < 24) were selected for the initial RNA-Seq profiling. A schematic of the study is given in Fig. 1.

**Table 1:** Details of the patient characteristics used in this study

Age - mean (SD)	70.66 (13.5)
Age at diagnosis - mean (SD)	68.02 (13.67)
BMI - mean (SD)	27.51 (4.91)
Post menopause	80%
Cancer type	Ductal (68.7%), lobular (21.7%), mucinous (3.6%), mixed (9.4%)
Cancer grade	Grade 1 (1.2%), Grade 2 (59%), Grade 3 (39.8%)
% ER receptor positive	87%
% HER2 receptor positive	18%
Family history	27%
Metastasis	43%
Lymph node positive	41%

### RNA isolation

Tissue biopsies (~ 100 mg) were obtained at the time of primary treatment (mastectomy surgery), before any other cancer treatments (such as chemotherapy or radiotherapy) and flash frozen in liquid N_2_ prior to RNA extraction. Isolation of total RNA including the miRNA fraction was carried out using 1 ml TRI Reagent® (Thermo Fisher, USA) supplemented with the addition of 10 mM MgCl2 to aid recovery of microRNAs [24]. Samples were then completely homogenised using a bead mill (Retsch Technology GmbH, Haan, Germany). Phase separation was carried out using chloroform. Total RNA was precipitated from the aqueous phase by means of an overnight incubation at − 20 °C with isopropanol. RNA pellets were then ethanol-washed twice and re-suspended in RNase-free dH_2_O. RNA quality and concentration was assessed by Nanodrop spectrophotometry (Wilmington USA).

### miRNA profiling by RNA-Seq

QC and quantity measurement of the RNA was performed on an Agilent 2100 Bioanalyzer. All RNA samples had OD260/280 ≥ 1.8 and RNA integrity number (RIN) ≥7. Selection of small RNAs for library preparation was performed using gel electrophoresis. Small RNA libraries were prepared using the NEB Next Multiplex Small RNA Library Prep Set for Illumina (New England Biolabs Cat. NEB-E7580S). The libraries were sequenced with one lane on an Illumina NextSeq 500 instrument with 50-bp single-end reads at the Genomics Center of the Silberman Institute of Life Sciences, Hebrew University, Jerusalem, Israel. Adaptor-only reads and low-quality reads were filtered out in the Illumina BaseSpace environment. RNA-seq datasets generated are available in the SRA repository (BioProject ID PRJNA494326). The raw dataset is available on https://www.ncbi.nlm.nih.gov/Traces/study/?acc=PRJNA494326.

### Quantification of miRNA and miR-10b target gene expression levels

Reverse transcription and quantitative PCR for candidate miRNAs were performed using TaqMan miRNA assays (Thermo Fisher, USA). A list of 22 experimentally validated targets of miR-10b was obtained from the validated miRNA target database, miRTarBase [25]. Reverse transcription of RNA was carried out using the Verso cDNA synthesis kit (AB1453B, Thermo Fisher, USA), according to manufacturer’s instructions, on the ABI-9600 platform. Target quantification was carried out on the Applied Biosystems ABI-7900HT Sequence Detection System platform using iTaq SYBR Green mix (172–5125, BioRad, Hercules, CA, USA) and DNA primers designed using Primer3 software. Primers were used at a final concentration of 1 μM and cycling conditions were according to the mix manufacturer’s instructions, in 4 technical replicates. All primers were tested for efficiency (by serial dilutions) and specificity (by melting peak analysis). Of the 22 targets selected, primer pairs for 15 targets passed QC as above (primers listed in Additional file 1: Table S2). Results were analyzed using the comparative Ct approach with subsequent global normalization in SDS 2.3 (Thermo Fisher, Warrington, UK) and Microsoft Excel software. Candidate microRNA levels and miR-10b target mRNA levels were correlated with BMI, age, cancer staging and other clinical parameters (see Table 1).

### Determination of miR-10b response to metabolic stimuli

Cultured BT-549 cells (purchased directly from ATCC, UK and passaged for fewer than 3 months) were treated with insulin (20 ng/ml), leptin (100 ng/ml) or linoleic, oleic, palmitoleic and palmitic acids (all at 0.3 mM), and stearic acid (0.1 mM) for 48 h. The dosage was determined based on published studies [26, 27] and by evaluating cytotoxicity. RNA was isolated using the Qiagen miRNeasy kit; miR-10b levels were measured by qRTPCR and compared to non-treated controls as described above.

### Manipulation of miR-10b levels and assessment of cell kinetics

Manipulation of miR-10b in cultured primary BC-derived cell line BT-549 was performed by lipophilic transfection of miR-10b-mimicking and antisense oligonucleotides (Dharmacon, Lafayette, CO, USA) and appropriate controls. Oligonucleotides and reagents were used according to manufacturer instructions*.* Transfection efficiency was measured by the TOX cytotoxic oligo transfection control (Dharmacon, Lafayette, CO, USA), and reached ~ 80%. RNA was isolated from cultured cells using the QIAZOL reagent (Qiagen, Paisley, UK). Transfected cells were assayed after 48 h for effects on growth, invasion and miR-10b target mRNA expression*.* Proliferation of cells was measured using the CyQUANT® Direct Cell Proliferation Assay (Life Technologies, Warrington UK) in 96-well plate format in a Tecan Infinite M200 Pro spectrophotometer. Invasion of cells was measured after 48 h using Matrigel™ (BD) in inserts (Greiner, Kremsmünster, Austria) placed in 24-well cell culture plates (Biological Industries, Beit HaEmek, Israel). Target gene expression was measured as described above.

### miR-10b promoter analysis

~ 12 kb of genomic sequence bordered by the miR-10b gene (on the plus strand) and the *HOXD-AS2* gene (on the minus strand) were analyzed using the Cister online tool [28] with default parameters.

### Statistics

RNA sequencing results were analyzed with Illumina BaseSpace using the small RNA application, CLCBio Genomics Workbench (version 7) using the default small RNA pipeline, and Microsoft Excel. Data were examined for statistical significance using Student’s t-tests and Pearson correlations as appropriate. Bonferroni correction for multiple testing was used where indicated. For the in vitro work, differences between miRNA expression levels under various test conditions were assessed by Student’s t-tests and Pearson correlations as appropriate.

## Results

### MicroRNAs miR-21, miR-451a, miR-10b, miR-30c-1, and miR-378d-2 show robust associations with cancer status in primary breast cancer samples

**Fig. 2:**
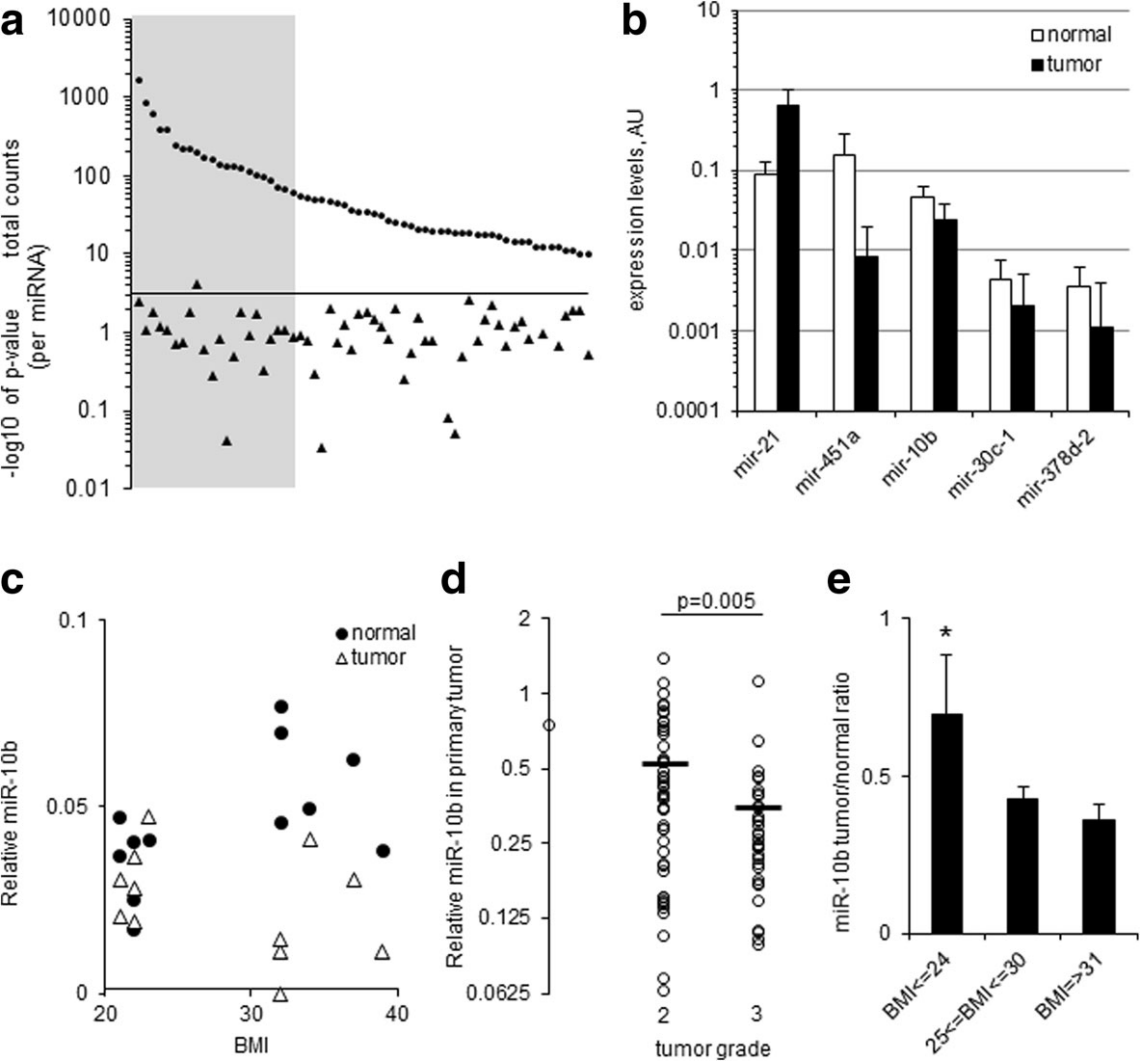
miR-10b shows stronger down-regulation in the tumors of overweight breast cancer patients. **a** The total counts (in paired 12 tumor and 12 normal tissue samples) of 62 miRNAs with 10 counts or more (round markers), and their significance of differential expression between normal and tumor samples expressed in –log_10_ of the paired T-test *p*-value (triangular markers). The grey area marks 22 highly-expressed miRNAs (with experimental range of 10 or more counts). The triangle above the line represents miR-10b with *p* = 7.6*10^− 5^. The horizontal axis is set at *p* = 0.0025 (Bonferroni correction for 22 miRNAs). **b** 5 miRNAs showing nominally significant changes between tumor and normal tissue in NGS analysis (*p* < 0.05, paired T-test, *N* = 6 for each group, 24 samples total). Note logarithmic scale of vertical axis. **c** Individual miR-10b expression levels, NGS analysis (as in (a)). **d** Relative miR-10b levels in tumor samples according to tumor grade, qRTPCR. (*N* = 83; horizontal bars represent averages; *p* = 0.005, t-test.) Note logarithmic scale of vertical axis. **e** Tumor/normal ratios of miR-10b expression levels, qRTPCR. * represents nominally significant differences between the leanest group and the most overweight group, and also between the leanest group and the rest of the cohort combined (*p* = 0.04 for both). Bars, standard errors; *N* = 19, 44 and 20 for the 3 groups respectively

To test if miRNAs are involved in the regulation of tumorigenesis-related gene expression in BC cells downstream of overweight/obesity, we profiled miRNA levels in paired tumor and normal tissue biopsies from 6 lean (BMI < 24) and 6 obese (BMI > 31) BC patients at the extremes of the cohort BMI distribution, using RNA-Seq. Between 10 M and 26 M reads were obtained per sample. Assembled transcripts numbered between 1 and 2 M per sample; of these, approximately 1% mapped to miRbase miRNAs. A total of 148 miRBase miRNAs were identified, of which 62 had a total of 10 or more specific mature reads in the 24 samples, and 22 had a normalized experimental range between 10 and 462 specific mature reads (Figure S2a and Additional file 2: Table S1). Of these, five miRNAs (miR-21, 451a, 10b, 30c-1, and 378d-2) showed nominally significant (*p* < 0.05) up- or down-regulation in the tumor tissue compared to normal tissue from the same patient (Fig. 2a, b). For miR-10b (*p* = 7.6X10^− 5^) the difference was still significant after applying the Bonferroni correction (based on 22 miRNAs).

### miR-10b demonstrates a larger deregulation in the primary tumors of overweight and obese BC patients compared with leaner women

Of the 5 miRNAs identified by RNA-Seq as differentially expressed in normal and tumor tissue, miR-10b was unique in that its levels were increased in the normal tissue of obese patients as compared to the lean, but downregulated in the tumors of the same obese patients (Fig. 2c). Measured by qRTPCR in all 83 sample pairs, tumor levels of miR-10b also showed a significant inverse correlation with tumor grade (Pearson correlation values of R = − 0.31, *p* = 0.004) and a significant difference between the grade 2 and grade 3 groups (*p* = 0.005) (Fig. 2d). The tumor/normal ratio of miR-10b levels also showed a significant inverse correlation with tumor grade (Pearson correlation values of R = − 0.25, *p* = 0.022). Furthermore, the down-regulation of miR-10b levels in tumors compared to the corresponding normal tissue was progressively stronger with higher BMI. This effect was statistically significant when comparing the leanest 19 subjects (BMI < =24) with the most obese 20 subjects (BMI= > 31), and also when comparing the leanest group as above with the rest of the cohort (*p* = 0.04 for both) (Fig. 2e). The levels of miR-30c in tumor tissue similarly showed a significant inverse correlation with tumor grade (Pearson correlation values of R = − 0.33, *p* = 0.002).

### miR-10b and its target mRNA levels show significant correlations with tumor grade

**Fig. 3:**
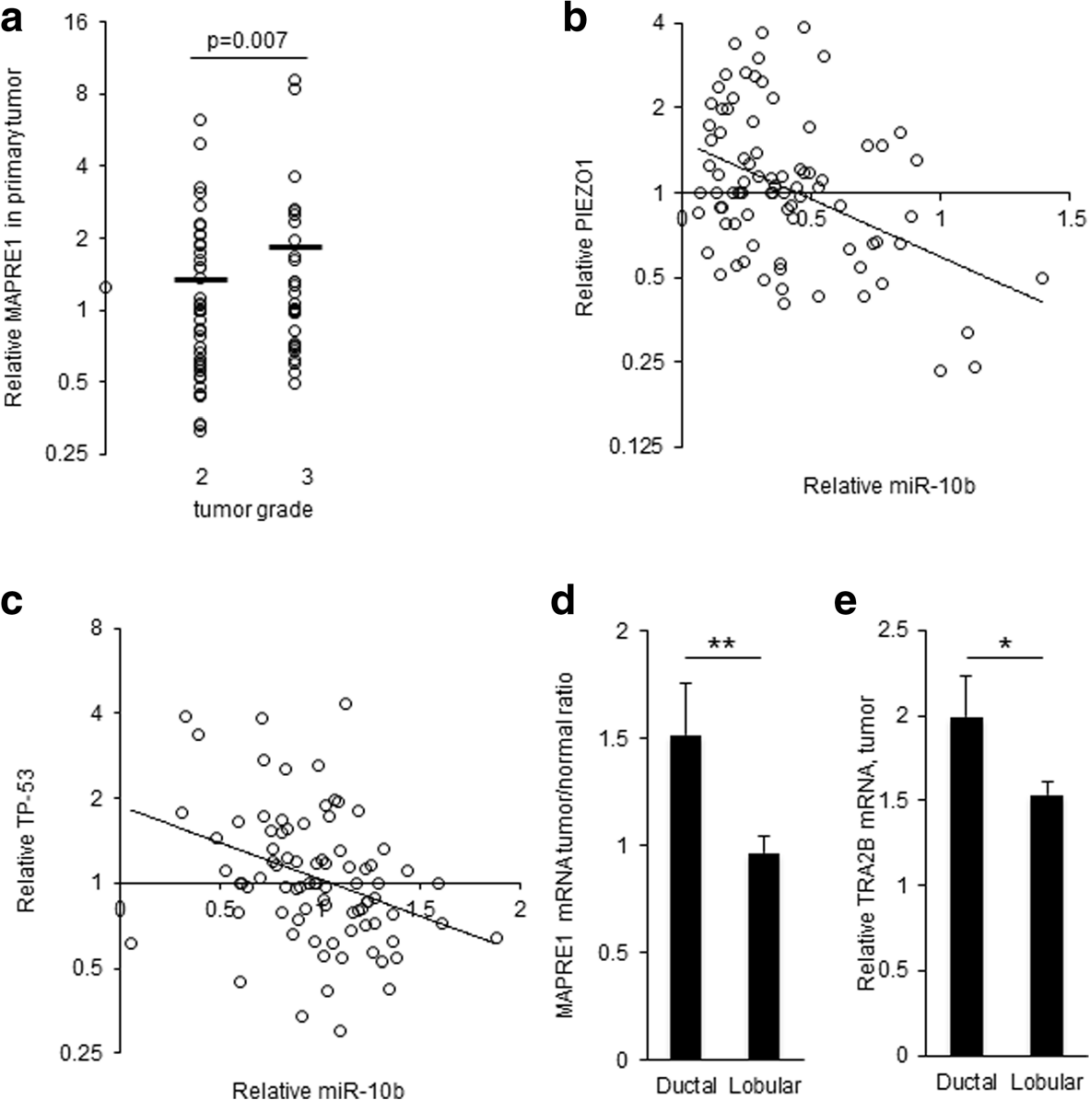
Cancer-relevant miR-10b target mRNAs show inverse correlation with miR-10b levels. **a** Relative MAPRE1 levels in tumor samples according to tumor grade, qRTPCR. (*N* = 83; horizontal bars represent averages; *p* = 0.007, t-test.) Note logarithmic scale of vertical axis. **b** Relative miR-10b levels plotted against relative PIEZO1 mRNA levels in normal breast tissue (*N* = 83; note logarithmic scale of vertical axis) **c** Relative miR-10b levels plotted against relative TP-53 mRNA levels in normal breast tissue (*N* = 83; note logarithmic scale of vertical axis). **d** Relative MAPRE1 levels in tumor samples according to tumor type, qRTPCR. (Ductal, *N* = 57; lobular, *N* = 18; error bars: std. err; **: p = 0.005; t-test.) **e** Relative TRA2B levels in tumor samples according to tumor type, qRTPCR. (Ductal, N = 57; lobular, *N* = 18; error bars: std. err; *: *p* = 0.05; t-test.)

To assess the physiological and pathological significance of the observed down-regulation of miR-10b, we measured by qRTPCR the mRNA levels of 15 previously validated targets of miR-10b (primer sequences in Additional file 1: Table S2) based on the validated miRNA target database, miRTarBase [25], in all the RNA samples from our 83-patient cohort. We identified that miR-10b levels showed a significant inverse correlation with target mRNA levels in at least one subset of samples (the tumor or the benign) for 4 out of 15 genes tested: *MAPRE1, PIEZO1, SRSF1 and TP53* (Fig. 3a-c). These cancer-relevant targets showed Pearson correlation values of − 0.276, − 0.298, − 0.265 and − 0.337 respectively, with *p*-values of 0.012, 0.006, 0.015 and 0.002 respectively. The correlation for TP-53 was significant after Bonferroni adjustment.

### Levels of miR-10b and its targets support greater role of miR-10b in ductal vs. lobular tumors

To check if the observed associations vary with tumor type, the samples were stratified by type (57 ductal carcinoma, 18 lobular carcinoma, and 8 mucinous or mixed which were excluded from further association analysis). In ductal carcinoma samples, significant inverse correlations were observed between tumor grade and the tumor levels of miR-10b and miR-30c (Pearson correlation values of R = − 0.26, *p* = 0.047 and R = − 0.39, *p* = 0.003, respectively). In contrast, the lobular carcinoma samples showed no significant correlations between tumor grade and the levels of these miRNAs. We also noted differences between ductal carcinomas and lobular carcinomas in terms of miR-10b mRNA target gene levels. Thus, target gene levels showed stronger associations with miR-10b and clinical measurements in ductal tumors as opposed to lobular tumors. In ductal carcinoma samples, significant inverse correlations were observed between tumor levels of miR-10b and tumor levels of six target mRNAs: *SRSF1* (R = − 0.26, p = 0.047), *PIEZO1* (R = − 0.26, *p* = 0.048), *MAPRE1* (R = − 0.28, *p* = 0.033), *CDKN2A* (R = − 0.29, *p* = 0.031), *TP-53* (R = − 0.371, *p* = 0.0045) and *TRA2B* (R = − 0.37, *p* = 0.009). In contrast, in lobular tumors no significant inverse correlations were observed between tumor levels of miR-10b and tumor levels of the measured miR-10b target mRNAs. Levels of *MAPRE1* and *TRA2B* mRNA were also higher overall, in ductal vs. lobular tumors (Fig. 3d-e).

### miR-10b promoter analysis

To look into the upstream regulation of miR-10b in the context of metabolic signaling, ~ 12 kb of genomic sequence bordered by the miR-10b gene (on the plus strand) and the *HOXD-AS2* gene (the nearest gene on the minus strand) were analyzed using the Cister online tool [28]. Predicted cis-elements were found on the plus strand for the following transcription factors: *NF-1, Sp1, Myf, Mef-2, ETS* and *SRF* (Additional file 1: Table S3 and Figure S1A).

### Manipulation of miR-10b levels in BT-549 primary BC cell line affects the expression levels of miR-10b target genes

**Fig. 4:**
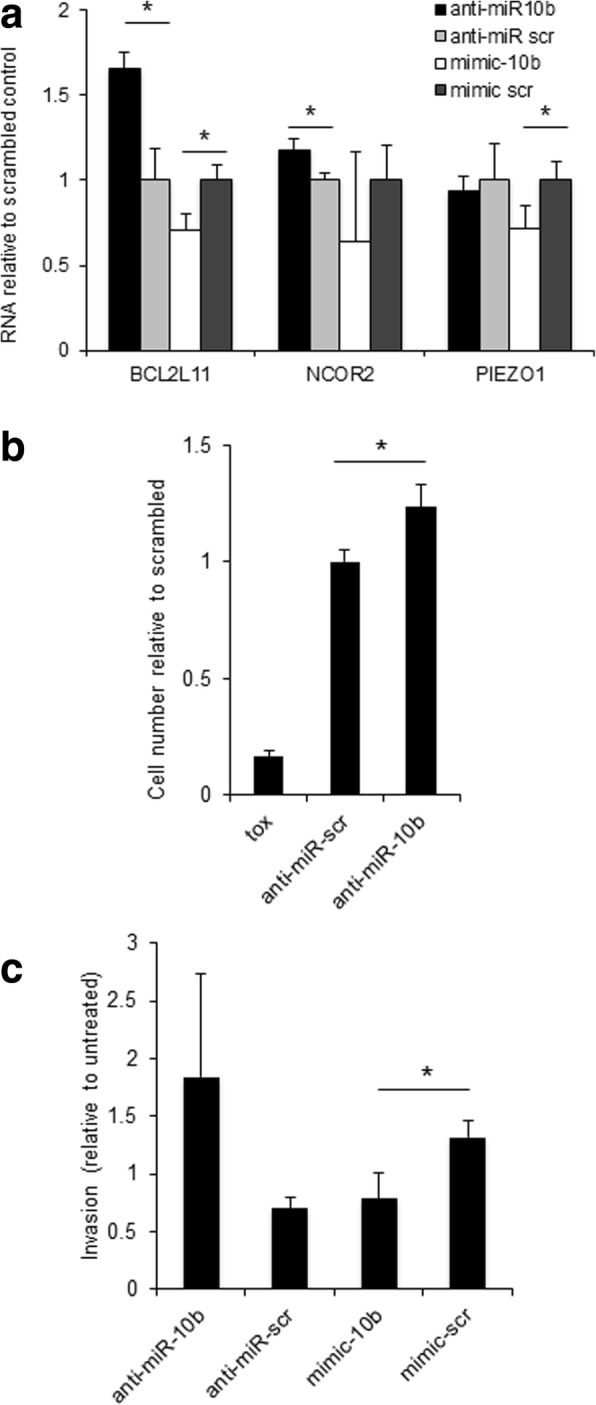
suppression of miR-10b in cultured BT-549 cells increases cell proliferation and invasion. **a** mRNA levels of miR-10b target genes (relative to the appropriate scrambled controls) in cultured BT-549 cells transfected with oligonucleotides as indicated, qRTPCR. Shown are average values from experimental triplicates. Bars, stdev. *:*p* < 0.05 (t-test). **b** Quantification (using the CyQUANT® Direct Cell Proliferation Assay) of BT-549 cell proliferation 48 h after transfection with anti-miR-10b oligos, scrambled controls, and the TOX oligo as transfection control. Background-subtracted signals were normalized to the scrambled control – transfected cells. Shown are averages from 9 or more biological replicates. Bars: standard errors. *: *p* = 0.023 (t-test). **c** Quantification (using the CyQUANT® Direct Cell Proliferation Assay) of BT-549 cell invasion through Matrigel™-coated inserts, 48 h after transfection with anti-miR-10b and miR-10b- mimicking oligos or the appropriate scrambled controls. Background-subtracted signals were normalized to non-transfected cells. Shown are averages from 3 biological replicates. Bars: standard errors. *: *p* = 0.017 (t-test)

To check if the exposure to metabolic hormones or free fatty acids can account for the differential expression of miR-10b in the tumors of lean and overweight BC patients, we treated a human primary breast cancer line, BT-549 cells with insulin (20 ng/ml), leptin (100 ng/ml), linoleic, oleic, palmitoleic and palmitic acids (all at 0.3 mM), and stearic acid (0.1 mM). We identified no effect of treatment with any of these factors on miR-10b expression levels (Additional file 1: Figure S1B, C). We then transfected BT-549 cells with anti-miR-10b oligos as well as scrambled controls, isolated the cells’ RNA and measured the mRNA levels of 15 previously validated targets of miR-10b (Additional file 1: Table S2) by qRTPCR. *BCL2L11, PIEZO1* and *NCOR2* were down-regulated in cells treated with miR-10b mimic, compared to a scrambled mimic control oligo (significantly for *BCL2L11* and *PIEZO1*). *BCL2L11* and *NCOR2* also showed a significant up-regulation in anti-miR-10b treated cells, compared to a scrambled anti-miR control (Fig. 4a).

### Suppression of miR-10b in cultured BT-549 cells increases cell proliferation and invasion

To elucidate the relevance of miR-10b down-regulation in primary breast cancer cells to cell proliferation, we transfected BT-549 cells with anti-miR-10b oligos as well as scrambled controls and measured cell proliferation after 48 h using the CyQUANT fluorescent assay. Transfection with anti-miR-10b oligo, which reached > 80% efficiency based on TOX oligo control, resulted in a 23% increase in cell proliferation compared to scrambled oligo control, which was statistically significant (*p* = 0.023) (Fig. 4b).

The effects of manipulating miR-10b levels on the invasiveness of primary breast cancer cells were also assessed by transfecting BT-549 cells with anti-miR-10b and miR-10b mimicking oligos as well as the appropriate scrambled controls and measuring cell invasion through Matrigel™- coated inserts after 48 h using the CyQUANT fluorescent assay. Transfection with anti-miR-10b oligo resulted in a ~ 2-fold increase in invasion compared to scrambled anti-miR control, which fell short of statistical significance due to high variability (*p* = 0.078) although invasion was higher in all replicate anti-miR-10b transfected samples. Conversely, transfection with miR-10b mimicking oligo resulted in a ~ 70% decrease in invasion compared to scrambled mimic control, which was statistically significant (*p* = 0.017) (Fig. 4c).

## Discussion

**Fig. 5:**
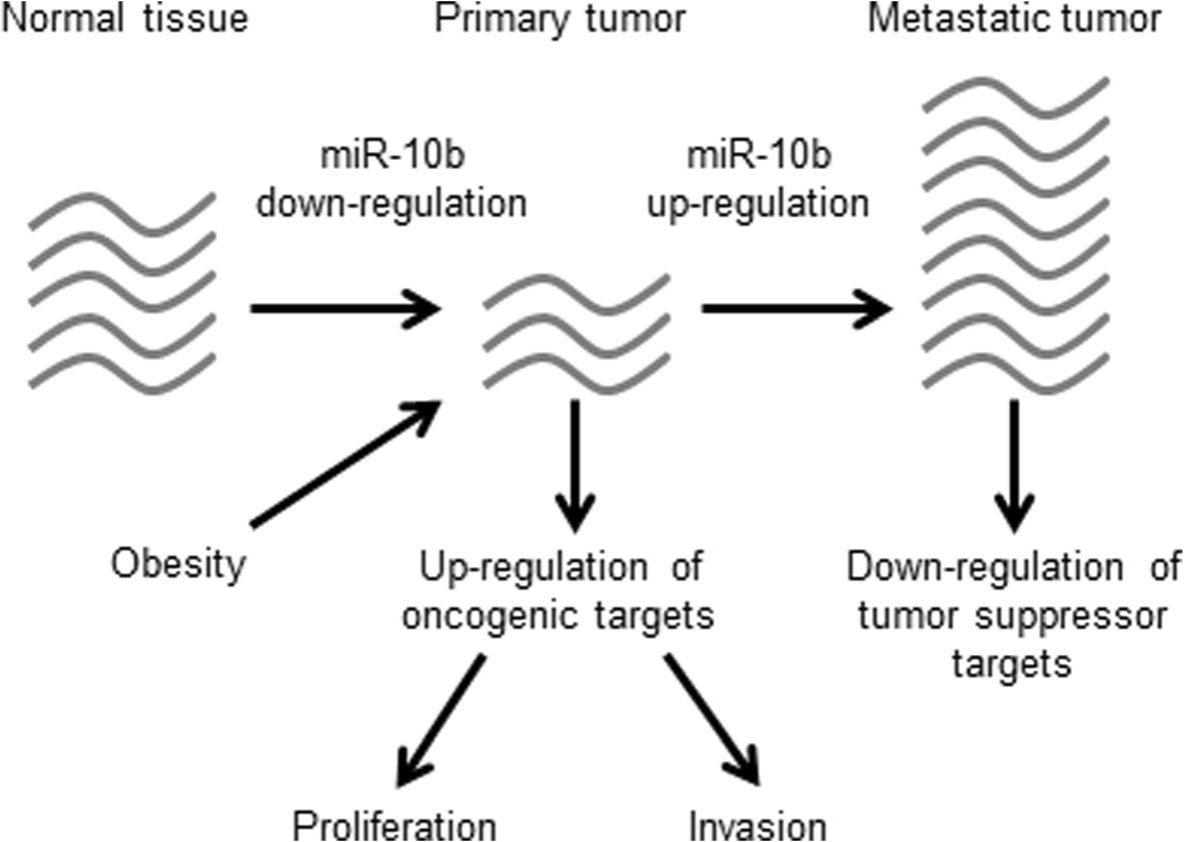
Summary scheme of suggested mechanism by which the down-regulation of miR-10b in the primary tumor, and then its up-regulation in the metastatic tumor are consecutively involved in breast cancer progression. The number of gray curves illustrates the relative levels of miR-10b. Obesity exacerbates the down-regulation of miR-10b in the primary tumor

In this study, we sought to test the hypothesis that miRNAs may function as a link between obesity and breast cancer, using total RNA from matched tumor and normal tissue biopsies from 83 BC patients. We identified five miRNAs (miR-21, miR-451a, miR-10b, miR-30c-1, and miR-378d-2) with altered expression between normal tissues and tumors. Of these, miR-21 is a well-known BC-related miRNA [29]. Although robust and significant, the up-regulation of miR-21 in tumors was independent of obesity, and has been extensively documented in the previous literature. MiR-10b was identified as a candidate miRNA for facilitating an interaction between tumorigenesis and obesity as its levels were affected by BMI. miR-10b is a member of a very ancient, highly conserved family of miRNAs well-characterized in humans and other species [30–32]. Its genomic locus is found inside the homeobox D cluster on Chromosome 2, and the *HOXD-AS2* antisense RNA gene is a close upstream neighbor, located about 12 kb away on the opposite strand. Promoter analysis suggests that these 2 RNA genes share enhancer sequences and may be co-regulated (Additional file 1: Figure S1A). The miR-10 family regulates several *HOX* genes as well as the *Wnt, Fgf* and *Notch* signaling pathways, as shown in mouse studies (review, [33]). Although the pathological role of miR-10b is still debated [32, 34, 35], miR-10b is known to be upregulated in metastatic BC cells and promote metastasis [30]. Accordingly, it has several validated targets that are involved in tumor growth and metastasis, and its role in these processes has already been described [31, 36–39]. However, a decrease in the levels of miR-10b in primary tumor cells, corresponding with the clinical progression of the disease, has previously been observed [40]; and this was supported by our findings. This apparent duality, namely the down-regulation of miR-10b in the primary tumor and then its up-regulation in the metastatic tumor, likely results from the diverse targets of this microRNA. Some of these targets are known tumor suppressors while others are oncogenes; as the specific selective pressure on their expression varies between tumor types and stages, so does the “optimal” level of miR-10b expression (scheme, Fig. 5). Any cell line used as an experimental model provides a limited “snapshot” of this complex process; not only due to its homogenous nature, but also due to a selective pressure under culture conditions, which is both constant and non-physiological. Furthermore, the expression of most genes is subject to combinatorial regulation on many levels (including multiple miRNAs), which is a likely explanation for the fact that the mRNA levels of most of the validated miR-10b targets tested did not show a significant negative correlation with miR-10b levels. Another possible explanation is that miRNA – mediated regulation of gene expression often has a more robust effect on the protein level rather than mRNA.

To date, the effects of manipulating miR-10b levels were described mostly in metastatic but not primary breast cancer cell lines. Our data show that suppression of miR-10b levels in BT-549 cells significantly increased cell proliferation after 48 h. Over longer periods of time, modest effects on proliferation can be magnified dramatically. The invasion capacity of BT-549 cells was also enhanced by the suppression of miR-10b levels and suppressed by transfection with a miR-10b-mimicking oligo. These changes in cell behavior parameters, which are hallmarks of cancer, were accompanied by altered mRNA levels of cancer-relevant direct targets of miR-10b: *BCL2L11, NCOR2* and *PIEZO1*. Taken together, these results provide evidence to the physiological significance of miR-10b down-regulation in primary breast tumors, as reported by our and other studies.

In a prior in vivo study in rats, the levels of miR-10b, among other miRNAs, were altered following a diet containing particular fatty acids [41]. In our in vitro data, however, no changes in miR-10b levels were observed in BT-549 cells treated with FFAs, leptin, or insulin. It is possible that the in vivo effect exists in humans as well, but is mediated by an indirect mechanism. For example, other cells or tissues beyond the primary tumor may respond to FFA levels by secretion of another signal molecule(s), which in turn may affect miR-10b expression in the tumor cells. The identity of this putative signal and its functions remain to be elucidated in ongoing studies, but it is likely to function via signaling pathways which include the transcription factors identified in the miR-10b promoter analysis.

In ductal but not lobular tumors miR-10b levels showed a significant inverse correlation with the mRNA levels of six previously validated miR-10b targets, as well as with tumor grade. Among these targets, *SRSF1* and *TRA2B* are key splicing activators, both involved in dysregulated splicing in breast cancer [24, 42, 43]. *PIEZO1* is a mechanosensory ion channel with oncogenic function in breast cancer, especially in metastasis [44] and has been examined as a potential therapeutic target [45]. *MAPRE1* is another oncogenic target of miR-10b, known to regulate microtubule dynamics and a proposed biomarker of gastric and colorectal cancer [46–48]. *CDKN2A* (*p16*) is important in cell cycle regulation and DNA repair, and mutations in it have been described as contributors to breast cancer risk [49, 50]. Finally, *TP-53* is one of the best-studied tumor suppressors, frequently mutated in breast cancers to produce inactive or oncogenic forms, with multiple functions in stress-induced processes such as DNA repair and apoptosis as well as in normal cell cycle regulation [51–53]. These findings suggest that the pathological role of miR-10b in ductal tumors is more significant than in lobular tumors; however, this hypothesis needs to be further tested in larger cohorts, as our sample contained only 18 patients with lobular tumors. The sample size (83 subjects) was, in general, a limitation of this study in that it restricted our ability to further stratify the data; the reproducibility of our findings should be tested in additional, and larger, patient cohorts.

## Conclusions

Our findings help elucidate the known duality of miR-10b regulation in breast cancer (down-regulation in primary tumors vs. up-regulation in metastases; scheme, Fig. 5). We found that obesity exacerbated the decrease in miR-10b in primary tumors compared to normal tissue, supporting the notion that the metabolic state of the organism can alter the molecular makeup of a tumor. Specifically, our findings suggest that altered miR-10b expression can differentially affect BC risk and progression based on overweight/obesity status, and may be useful in diagnosis, prognosis, and personalization of BC therapy.

## Additional files

Additional file 1: **Table S2.** List of primers used for qRTPCR of miR-10b target genes. **Table S3.** Cister cluster prediction for 12 kb interval between miR-10b and HOXD-AS2 genes (predicted cis-element table). **Figure S1.** A. Cister cluster prediction for 12 kb interval between miR-10b and HOXD-AS2 genes (graph). B-C: qRT-PCR for miR-10b, miR-21 and miR-451a levels in BT-549 cells treated with hormones (B) or free fatty acids (C). (DOCX 27 kb)

Additional file 2: **Table S1.** miRbase miRNAs identified in RNA-seq. The first worksheet lists all miRbase entries identified in 24 samples (normal + tumor) from 12 subjects, with individual values. The second worksheet is an abbreviated list including only the robustly expressed miRNAs (normalized range > 10 reads/sample) with group statistics. (XLSX 577 kb)

## Abbreviations

BC: Breast cancer
BMI: Body mass index
FFA: Free fatty acids
miRNA: microRNA
qRTPCR: Reverse transcription and quantitative polymerase chain reaction
RNA-Seq: RNA sequencing
