# Supplementary material for: Obesity impacts the regulation of miR-10b and its targets in primary breast tumors
Source: BMC Cancer. 2019 Jan 18;19:86. doi: 10.1186/s12885-019-5300-6 (PMC6339293; doi:10.1186/s12885-019-5300-6)
Supplement: Supplementary file 1 — Table S2. List of primers used for qRTPCR of miR-10b target genes. Table S3. Cister cluster prediction for 12 kb interval between miR-10b and HOXD-AS2 genes (predicted cis-element table). Figure S1. A. Cister cluster prediction for 12 kb interval between miR-10b and HOXD-AS2 genes (graph). B-C: qRT-PCR for miR-10b, miR-21 and miR-451a levels in BT-549 cells treated with hormones (B) or free fatty acids (C). (DOCX 27 kb) [file 12885_2019_5300_MOESM1_ESM.docx]

**Supplementary Table 2**: list of primers used for qRTPCR of miR-10b target genes

|  | **FWD** | **Rev** |
| --- | --- | --- |
| **BCL2L11** | ATCCCCGCTTTTCATCTTTA | AGGACTTGGGGTTTGTGTTG |
| **BDNF** | AGTGCCGAACTACCCAGTCGTA | CTTATGAATCGCCAGCCAATTC |
| **CDKN1A** | GTACCACCCAGCGGACAAGT | CCTCATCCCGTGTTCTCCTTT |
| **CDKN2A** | CATAGATGCCGCGGAAGGT | CCCGAGGTTTCTCAGAGCCT |
| **HOXD10** | GGGGACCTATGGAATGCAAAC | CGGATCTGTCCAACTGTCTACT |
| **KLF4** | ACCAGGCACTACCGTAAACACA | GGTCCGACCTGGAAAATGCT |
| **MAPRE1** | GGCTCCTTCCCTTGTTGCTC | CGTCTCCGTTGCCCACAC |
| **NCOR2** | GGTCAAGTCCAAGAAGCAAGAGAT | GCTTCTATAGGTCATAAGGCCTGTTC |
| **PAX6** | CCGTGTGCCTCAACCGTA | CACGGTTTACTGGGTCTGG |
| **PIEZO1** | TGAAGCGGGAGCTCTACAAC | TCTCGTTGGCATACTCCACA |
| **PPARa** | GGCGAGGATAGTTCTGGAAGC | CACAGGATAAGTCACCGAGGAG |
| **PTEN** | CCGAAAGGTTTTGCTACCATTCT | AAAATTATTTCCTTTCTGAGCATTCC |
| **SRSF1** | CTTGGTGGGAAGGCCTGTT | AGATGCGGCAATCGTTGTTC |
| **TP53** | TCAACAAGATGTTTTGCCAACTG | ATGTGCTGTGACTGCTTGTAGATG |
| **TRA2B** | TAGGCGTTCAAGAGGATTTG | TTCCATTGGCACGTTCTTTA |

**Supplementary Table 3**: Cister cluster prediction for 12kb interval between miR-10b and HOXD-AS2 genes (predicted cis-element table).

| **type** | **position** | **strand** | **sequence** | **probability** |
| --- | --- | --- | --- | --- |
| NF-1 | 2920 to 2937 | + | ctttggctacatttccaa | 0.3 |
| Sp1 | 2784 to 2796 | + | tgggggcgggggg | 0.26 |
| LSF | 7632 to 7646 | - | ctagtgctaaccaga | 0.21 |
| Myf | 5575 to 5586 | + | gggcagcagctg | 0.2 |
| GATA | 2944 to 2956 | - | tgacttatcacct | 0.18 |
| Mef-2 | 5627 to 5638 | + | gggtatttttat | 0.18 |
| ERE | 7500 to 7513 | - | ggtcaggctggtct | 0.17 |
| NF-1 | 5608 to 5625 | - | tgggggttgtagccagaa | 0.16 |
| Ets | 2820 to 2830 | + | agcaggaagat | 0.14 |
| SRF | 7698 to 7710 | + | aaccataaaagaa | 0.14 |
| SRF | 3287 to 3299 | + | ggacatataaggg | 0.14 |
| SRF | 7736 to 7748 | + | aacaaaaaaagga | 0.14 |
| Tef | 3210 to 3221 | + | cacattcgaggg | 0.13 |
| Sp1 | 5506 to 5518 | - | agccccacctcca | 0.12 |
| NF-1 | 3151 to 3168 | - | ccacacttcaggccaatc | 0.11 |
| Sp1 | 6025 to 6037 | - | gccccctcccctt | 0.1 |
| Ets | 7809 to 7819 | + | atcaggaaatg | 0.1 |

**Supplementary Figure 1:** A. Cister cluster prediction for 12kb interval between miR-10b and HOXD-AS2 genes (graph). B-C: qRT-PCR for miR-10b, miR-21 and miR-451a levels in BT-549 cells treated with hormones (B) or free fatty acids (C) as indicated. Bars: stdev. From triplicates. None of the treatments led to statistically significant changes in measured miRNA levels.

**
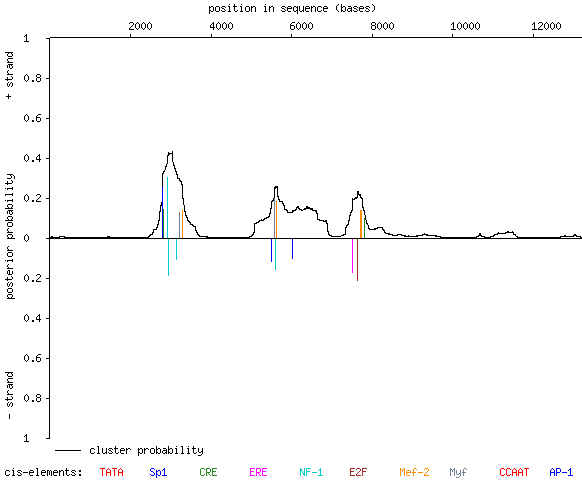
A**

**B**

**C**
